# CD137 ligand activated microglia induces oligodendrocyte apoptosis via reactive oxygen species

**DOI:** 10.1186/1742-2094-9-173

**Published:** 2012-07-16

**Authors:** Yee Andy Yeo, Julia M Martínez Gómez, J Ludovic Croxford, Stephan Gasser, Eng-Ang Ling, Herbert Schwarz

**Affiliations:** 1 Department of Physiology, Yong Loo Lin School of Medicine, National University of Singapore, Centre for Translational Medicine, 14 Medical Drive #14-02T, Singapore, 117599, Singapore; 2 Department of Microbiology, Yong Loo Lin School of Medicine, National University of Singapore, Block MD4, 5 Science Drive 2, Singapore, 117597, Singapore; 3 Department of Anatomy, Yong Loo Lin School of Medicine, National University of Singapore, Blk MD10, 4 Medical Drive, Singapore, 117597, Singapore; 4 Immunology Programme, National University of Singapore, Singapore, Singapore; 5 NUS Graduate School for Integrative Sciences and Engineering, National University of Singapore, Singapore, Singapore

**Keywords:** Microglia, Oligodendrocyte, Apoptosis, CD137, Experimental autoimmune encephalomyelitis, Multiple sclerosis

## Abstract

CD137 (4-1BB, TNFRSF9), a member of the tumor necrosis factor (TNF) receptor family, is a potent T cell co-stimulatory molecule. CD137 ligand (CD137L) is expressed by antigen presenting cells (APC) as a transmembrane protein and transmits activating signals into APC. In this study we investigated the effects of CD137L signaling in microglia, the resident APC in the central nervous system. *In vitro*, the murine microglia cell lines BV-2 and N9, as well as primary murine microglia responded with activation as evidenced by adherence and secretion of proinflammatory cytokines, MMP-9, and soluble intercellular adhesion molecule (ICAM). CD137L signaling is also important for microglia activation *in vivo*, since CD137L-deficient mice exhibited profoundly less microglia activation during experimental autoimmune encephalomyelitis (EAE) which is a well-established murine model for neuroinflammation and human multiple sclerosis (MS). Also CD137 is expressed in the CNS of mice during EAE. Activated microglia has been reported to mediate the destruction of axonal myelin sheaths and cause the death of oligodendrocytes, the main pathogenic mechanisms in EAE and MS. Corresponding to the lower microglia activation there were also fewer apoptotic oligodendrocytes in the CNS of CD137L-deficient mice. *In vitro* co-culture confirmed that CD137L-activated microglia induces apoptosis in oligodendrocytes, and identified reactive oxygen species as the mechanism of apoptosis induction. These data demonstrate activating effects of CD137L signaling to microglia, and show for the first time that the CD137 receptor/ligand system may be a mediator of neuroinflammatory and neurodegenerative disease, by activating microglia which in turn kill oligodendrocytes.

## Background

Multiple sclerosis (MS) is a severe autoimmune disease of the central nervous system (CNS), characterized by the loss of axonal myelin sheaths that leads to neuronal degeneration and subsequently to neurological and behavioral abnormalities. A major problem with MS is that we do not know its cause and that we only poorly understand its pathogenesis. A considerable amount of MS research is being carried out using experimental autoimmune encephalomyelitis (EAE), a murine model of neuroinflammation that mimics some aspects of MS [1,2].

CD137 (TNFRSF9, 4-1BB, induced by lymphocyte activation, ILA) is a member of the tumor necrosis factor (TNF) receptor family which is expressed on activated T cells, NK cells, and vascular endothelial cells and delivers potent co-stimulatory signals upon activation [3-6].

CD137L is expressed by APC, and APC use CD137L to co-stimulate CD137-expressing, activated T cells. The CD137 receptor/ligand system is capable of bidirectional signaling, a property it shares with several other members of the TNF receptor/ligand families [7]. The molecular basis of bidirectional signaling is that CD137L, just as CD137, is expressed as a transmembrane protein on the cell surface and that it can transmit a signal into the cell it is expressed on, a process referred to as reverse signaling [8].

Reverse signaling by CD137L activates peripheral monocytes evidenced by stronger adhesion, secretion of proinflammatory cytokines [9,10], increased survival [11], proliferation [12], and enhanced migration [13]. Further, CD137L signaling induces differentiation of monocytes to dendritic cells (DC) and DC maturation [14-16]. Even hematopoietic progenitor cells are stimulated by CD137L signaling which respond with proliferation and myeloid differentiation [17,18]. These data identify CD137L as potent growth factor for myeloid cells. Therefore, we hypothesized that CD137L signaling may also activate microglia which are the resident APC of myeloid origin in the CNS [19,20].

Our study confirms this hypothesis. CD137L signaling activates microglia cell lines and primary microglia cells *in vitro* leading to enhanced adhesion and secretion of proinflammatory cytokines. CD137L is also important for microglia activation *in vivo*, as its absence in genetically modified mice results in lower microglia activation during EAE. A key event in the pathogenesis of EAE and MS is the destruction of oligodendrocytes and their axonal myelin sheaths by activated immune cells. CD137L-activated microglia indeed induces apoptosis in oligodendrocytes and this cell death is mediated by reactive oxygen species (ROS).

## Methods

### Culture of murine microglia cell lines

The murine microglia cell line BV-2 was purchased from Banca Biologica e Cell Factory (IST Genova, Italy). BV-2 cells were cultured in Dulbecco’s Modified Eagle Medium (DMEM) high glucose medium (Sigma) containing 10% fetal bovine serum (Biowest) at 37°C in 5% CO_2_. Growth medium was changed every 3 to 4 days before cells reached confluence. The N9 cell line was a gift from Dr Wong Siew Heng from the Department of Microbiology, NUS. DMEM high glucose medium (Sigma) containing 10% fetal bovine serum (Biowest) culture medium was also employed for the culture of the murine N9 cell line. Growth medium was changed every 2 to 3 days until confluence was reached.

### Primary microglia culture

Six-to-eight-week-old female C57BL/6 mice were deeply anaesthetized with pentobarbitone (90 mg/kg). Transcardial perfusion was performed using 0.9% NaCl with ice cold heparin. Brain and spinal cord tissues were extracted and processed using the MACS Neural Tissue Dissociation kit and the GentleMACS Tissue Dissociator (Miltenyi). Manual cell count was performed using trypan blue staining, and the cells were resuspended in DMEM high glucose containing 10% fetal bovine serum (FBS), 1 x penicillin streptomycin (Life Technologies) and 10 ng/mL M-CSF (Peprotech). A seeding density of 1.5 x 10^6^ cells per mL was employed and cells were incubated for up to 4 days at 37°C in 5% CO_2_. Growth medium with 10 ng/mL M-CSF was supplied every 3 to 4 days for up to 4 weeks.

### Induction of CD137L signaling

Tissue culture plates were coated with PBS, 10 μg/mL of human Fc fragment (Millipore, Temecula, CA, USA) or 10 μg/mL human CD137-Fc (R&D Systems) overnight at 4°C. Then the wells were rinsed and cells were plated.

For microglia and OLN93 co-cultures, either primary microglia or BV-2 or N9 cells were co-cultured with OLN93 cells on PBS-, Fc-, and CD137-Fc-coated plates overnight. Murine microglia cells were labeled with 2 μM carboxyfluorescein succinimidyl ester (CFSE), (Invitrogen) prior to co-culture. A total of 10,000 U/mL of catalase were added to designated wells.

### Antibodies and flow cytometry

Phycoerythrin (PE)-conjugated and unconjugated anti-mouse CD137 antibody (clone 17B5) and anti-mouse CD137 ligand antibody (clone TKS-1) were obtained from eBioscience (San Diego, CA, USA). PE or fluorescein isothiocyanate (FITC) labeled rat anti-mouse CD11b, CD45, and respective isotype controls (rat IgG2a, rat IgG2b, Armenian Hamster IgG) were purchased from eBioscience. Non-specific staining was controlled by isotype-matched antibodies. For assessment of apoptosis, cells were stained with 7-AAD (BD Pharmingen) and Annexin V Alexa Fluor 647 (BioLegend). Flow cytometry was performed either on a FACSCalibur (BD Biosciences, San Jose, CA) with CellQuest data acquisition and analysis software, or on a Cyan flow cytometer (Dako, Denmark) with Summit software.

### Photographs

Morphological changes of cells were documented using a Zeiss Axiovert 40 inverted microscope (Zeiss, Göttingen, Germany) and Canon PowerShot G6 digital camera.

### Phagocytosis assay

Fifty yellow-green fluorescent latex beads of 1 μm diameter (FluoSpheres®, Molecular Probes) per cell were added to samples and incubated for 1 h at 37°C. Subsequently, phagocytosis was stopped by the addition of ice-cold PBS and cells were washed and treated with trypsin to dislodge any surface adherent latex beads. Cells were then resuspended in 400 μL PBS and flow cytometry was performed to quantify phagocytosis.

### ROS measurements

After 24 h of culture in wells coated with PBS, Fc, or CD137-Fc the cells were stimulated with 0.4 μg/mL PMA treatment for 1.5 h, and then stained with 100 ng/mL of dihydrorhodamine (DHR123, Invitrogen) for 25 min at 37°C. Then cells were washed to remove excess DHR123. ROS production was quantified using the FITC channel on a Cyan flow cytometer.

### ELISA

ELISA assays for TNF, sICAM-1, and Total MMP-9 (R&D Systems), and MCP-1, IL-1β, IL-6, and IL-12 p40 (Peprotech) were performed according to the manufacturers’ protocols. All measurements were performed in triplicates.

### EAE induction

All institutional guidelines for animal care and use were strictly adhered throughout the experiments. C57BL/6 mice were obtained from the Centre for Animal Resources (CARE) of the National University of Singapore, and CD137L^-/-^ mice were a gift from Amgen and bred in-house under pathogen-free conditions. Mice were injected subcutaneously with 100 μg of myelin oligodendrocyte glycoprotein peptide fragment 35-55 (MOG_35-55_) (Sigma-Aldrich) and 1 mg heat-killed Mycobacterium tuberculosis H37RA (Difco) emulsified in complete Freund’s adjuvant. Pertussis toxin (200 ng in PBS; List Biological Laboratories) was injected intraperitoneally on days 0 and 2 after immunization. EAE clinical symptoms were scored daily as follows: 0, no clinical signs; 1, loss of tail tonicity; 2, impaired righting reflex; 3, partial hind limb paralysis; and 4, total hind limb paralysis.

### Immunohistochemistry (IHC)

Transcardial perfusion was performed prior to sacrificing the mice. CNS tissues from naive and EAE mice were extracted and fixed with 10% neutral buffered formalin for 3 days. The tissues were paraffin-embedded and serially sectioned at 5 μM thickness (Leica Microsystems). After deparaffinization in Histo-Clear, and hydration in a graded series of alcohol, the slides were pretreated with citrate buffer (Dako) in a pressure cooker at 109°C for 20 min for antigen retrieval. Unspecific staining was blocked by 2% serum for 30 min. Endogenous peroxidases were inactivated by 3% hydrogen peroxide for 15 min. Anti-CD137 (goat polyclonal, R&D Systems) and anti-Iba-1 (rabbit polyclonal, Wako Chemicals) in PBS were used as primary antibodies and hybridized overnight. The secondary Strepavidin-HRP (Sigma) was added for 1 h, followed by DAB^+^ substrate (Dako). The entire procedure was carried out at room temperature, and after each step the samples were washed three times using PBS with 0.05% Tween 20. The tissue sections were counterstained with hematoxylin and excess counterstain was washed off with distilled water. Finally, the stained slides were dehydrated in a graded series of alcohol followed by Histo-Clear and mounted.

### TUNEL and immunofluorescence stainings

Terminal deoxynucleotidyl transferase dUTP nick end labeling (TUNEL) assay (Genscript) was performed on murine WT and CD137L^-/-^ CNS tissues according to manufacturer’s instructions. Oligodendrocytes, activated microglia, and nuclei were visualized with cyanine (Cy)3-conjugated anti-Nogo-A (sheep polyclonal, R&D Systems), Cy5-conjugated anti-Iba1 (rabbit polyclonal, Wako Chemicals), and DAPI, respectively.

### Immunofluorescence quantification

The Metamorph NX software (Molecular Devices, Sunnyvale, CA, USA) was employed for the quantification of Cy5-positive activated microglia as well as TUNEL FITC and Cy3 double-positive dead oligodendrocytes in WT and CD137L^-/-^ CNS. Cells between 8 and 30 μm in width with intensity of more than 10 units above background were gated. The data are presented as means ± standard deviations of measurements recorded from three to four separate fields across serial tissue sections, representing three independent experiments.

### Statistical analysis

Data are presented as means ± standard deviations. Student’s *t*-test was used to determine significant differences between control and treated groups. *P* <0.05 was regarded as statistically significant.

## Results

### Microglia express CD137L and become activated by CD137L signaling

**Figure 1:**
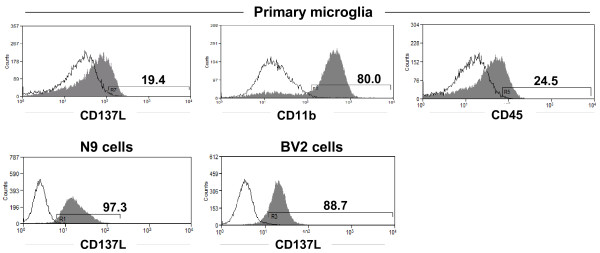
**Expression of CD137L on microglia.** Expression of CD137L on the murine microglia cell lines N9 and BV-2 and on C57BL/6 primary murine microglia was determined by flow cytometry. Open histogram: Isotype control. Grey histogram: Anti-CD137L monoclonal antibody (clone TKS-1). Primary microglia was also stained for CD11b and CD45. Numbers in panels indicate the percentages of CD137L^+^ cells.

Our preliminary data lend support to this hypothesis. CD137L is expressed on primary microglia and on the microglia cell lines BV-2 and N9. The myeloid nature of primary microglia was confirmed by staining for CD11b and CD45 (Figure 1).

Further, CD137L signaling activates microglia. CD137L signaling in microglia cells was induced with a recombinant CD137 protein that consists of the extracellular domain of CD137, fused to the constant domain of human IgG1 (Fc). This CD137-Fc fusion protein was immobilized on tissue culture plates to allow it to crosslink CD137L and thereby induce CD137L signaling in the microglia cells. Uncoated plates (PBS) or plates coated with Fc protein were used as negative controls.

**Figure 2:**
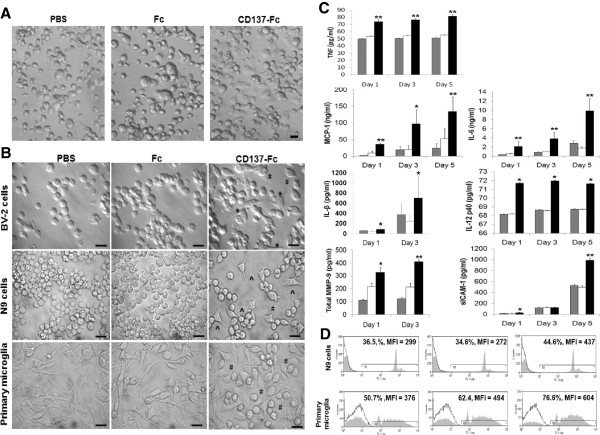
**CD137L signaling activates microglia*in vitro*.** Microglia cells were grown on plates that had been coated with PBS (grey bars) or 10 μg/mL of Fc control protein (white bars) or 10 μg/mL of CD137-Fc protein (black bars). (**A**) Attachment and morphological changes of primary microglia were documented by photography (40× magnification) 1 h after plating. Scale bar: 20 μm. (**B**) Attachment and morphological changes of BV-2 and N9 cells and primary microglia were documented by photography (63×) 24 h after plating. Cells exposed to immobilized CD137-Fc protein developed long spiky projections* and became amoeboid cells^ with shortened protrusions^#^. Scale bar: 20 μm. (**C**) The concentrations of cytokines in supernatants of primary microglia were determined by ELISA at indicated time points. Depicted are means ± standard deviations of triplicate measurements. (**D**) The phagocytic capacity of the cells was determined by adding FITC-labeled latex beads for 1 h before analysis by flow cytometry. Control: Autofluorescence of the cells. Numbers above the histograms state the percentages of positive cells and mean fluorescence intensities (MFI). These experiments were repeated three times with similar results. * *P* <0.05; ** *P* <0.01.

The activation of microglia by CD137L signaling was reflected by morphological changes such as increased adherence and cell spreading. The attachment of primary microglia on tissue culture plates was already visible after 1 h of seeding in the presence of CD137-Fc but not under the PBS and Fc control conditions (Figure 2A). Morphological changes of BV-2, N9, and primary microglia were evident a day after CD137L signaling (Figure 2B).

Further, CD137L engagement induced the secretion of proinflammatory cytokines (TNF, IL-1, IL-6, IL-12, MCP-1) in the two microglia cell lines BV-2 and N9 (not shown), and in primary microglia (Figure 2C). Also, matrix metalloproteinase (MMP)-9 and soluble ICAM were released by primary microglia in response to CD137L signaling (Figure 2C) and the phagocytic capacity was increased (Figure 2D).

### CD137L is required for microglia activation during neuroinflammation

**Figure 3:**
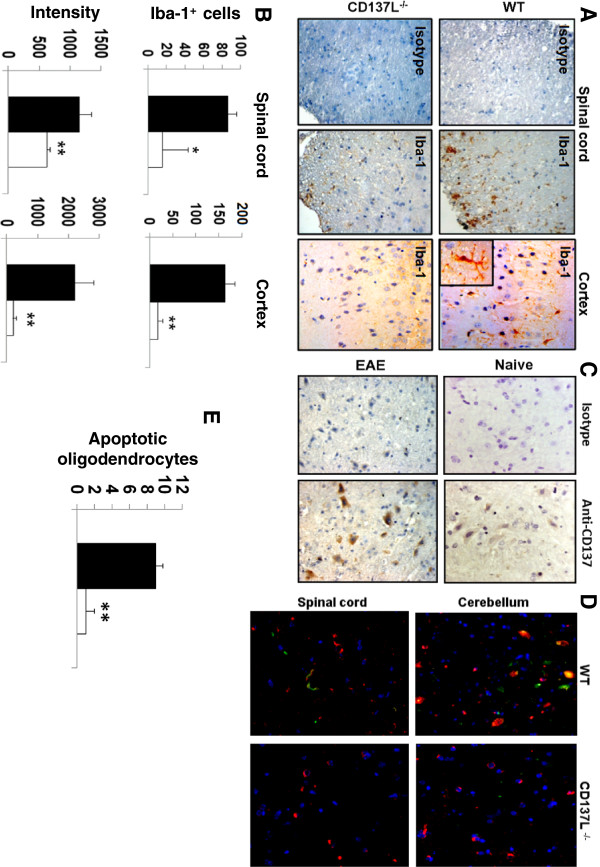
**CD137L signaling activates microglia*in vivo*.** (**A**) CD137L is required for activation of microglia *in vivo*. Cortex and spinal cord tissue sections of WT and CD137L^-/-^ mice with EAE were immunohistochemically stained with an isotype control antibody or for Iba-1 (brown). Shown in the inset is a close-up of a single Iba-1-positive microglia cell in the cortex of a WT mouse with EAE. (**B**) Quantification of Iba-1^+^ microglia in the spinal cords and cortices of WT and CD137L^-/-^ mice with EAE. Evaluated were three fields from two sections each using the Metamorph NX Software. Depicted are means ± standard deviations. (**C**) CD137 is expressed in the CNS during EAE. Spinal cord of naïve WT mice and WT mice with EAE was sectioned and stained with an isotype control antibody or for CD137 (brown). (**D**) The presence of CD137L is required for oligodendrocyte apoptosis in EAE. Tissue sections from the dorsal column of the spinal cord and the white matter of the cerebellum of WT and CD137L^-/-^ mice with EAE were stained for oligodendrocytes using a Cy3-labeled anti-Nogo-A antibody (red). Apoptosis was detected by TUNEL staining (green). Nuclei were visualized by DAPI (blue). The yellow staining results from an overlay of red and green and indicates apoptotic oligodendrocytes. Magnification: 40×. (**E**) Quantification of apoptotic oligodendrocytes in the cerebellum of WT and CD137L^-/-^ mice with EAE. Evaluated were three fields from two sections each using the Metamorph NX Software. Depicted are means ± standard deviations.

After having demonstrated *in vitro* that CD137L signaling activates microglia we aimed to confirm these data *in vivo*. For that we employed EAE, a murine model of neuroinflammation and human MS. These studies indicate that CD137L is not only able to activate microglia but seems to be essential. The brain cortices and dorsal columns of the spinal cords of WT mice with EAE exhibited strong Iba-1 expression whereas little or no Iba-1 expression could be detected in corresponding tissues of CD137L^-/-^ mice with EAE (Figure 3A). In the CD137L^-/-^ mice number of Iba-1^+^ cells as well as the staining intensity were significantly reduced (Figure 3B) indicating that limited redundancy exists to replace the CD137L signal.

But the absence of CD137L in the CNS of CD137L^-/-^ mice could only influence the development of EAE if also CD137 would be present. Indeed, immunohistochemical staining for CD137 confirmed its expression in the CNS, and expression increased during EAE (Figure 3C).

### CD137L-activated microglia induces apoptosis in oligodendrocytes

Microglia has been shown to play a pivotal role in EAE and MS [21,22]. The major pathogenic event in MS is the killing of oligodendrocytes by activated immune cells, including microglia, leading to the loss of axonal myelin sheaths and the subsequent death of the affected neurons [23]. Therefore, we investigated the frequency of apoptotic oligodendrocytes in the CNS of WT and CD137L^-/-^ mice with EAE by staining for apoptosis-specific DNA fragmentation and Nogo-A, an oligodendrocyte marker. The absence of CD137L, which was associated with much lower microglia activation also resulted in a lower number of oligodendrocytes undergoing apoptosis in the dorsal column of the spinal cord as well as the white matter of the cerebellum (Figure 3D). For example, nine times more apoptotic oligodendrocytes could be detected in the cerebella of WT mice compared to CD137L^-/-^ mice during EAE (Figure 3E).

The pathogenesis of EAE in the CD137L^-/-^ mice shows that reduced microglia activation is associated with a reduced oligodendrocyte death. In order to provide evidence that the reduced microglia activation is responsible for the reduced oligodendrocyte death we tested whether CD137L-activated microglia affect the viability of oligodendrocytes.

**Figure 4:**
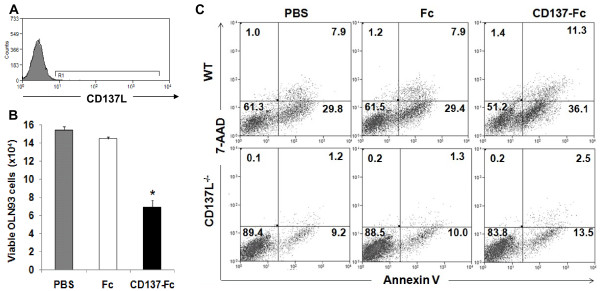
**Induction of oligodendrocyte death by CD137L-activated microglia. (A**) Expression of CD137L on the oligodendrocyte cell line OLN93 was determined by flow cytometry. Open histogram: Isotype control. Grey histogram: Anti-CD137L monoclonal antibody (clone TKS-1). (**B**) N9 and OLN93 cells were cultured for 24 h at a 1:1 ratio (1.5×10^5^ each) on plates that had been coated with nothing (PBS) or 10 μg/mL of Fc control protein or 10 μg/mL of CD137-Fc protein. (**C**) The rates of OLN93 cell apoptosis in co-cultures with primary microglia from WT or CD137L^-/-^ mice was determined 48 h after initiation of CD137L signaling by 7-AAD and Annexin V staining. Numbers in quadrants indicate percentages of cells. These experiments were repeated two to three times with similar results. * *P* <0.05; ** *P* <0.01.

In contrast to microglia, CD137L is not expressed by the oligodendrocyte cell line OLN93 (Figure 4A) and could also not be detected on primary oligodendrocytes (not shown). Accordingly, treatment of OLN93 cells with recombinant CD137-Fc protein had no effect on cell numbers and the rate of apoptosis (not shown).

When CD137L-activated N9 microglia cells were co-cultured with OLN93 oligodendrocytes the number of viable OLN93 cells was significantly reduced compared to OLN93 cells that had been co-cultured with unactivated microglia or microglia that had been treated with the Fc control protein (Figure 4B). This decrease in the live OLN93 cells was also demonstrated with CD137L-activated primary microglia, and was accompanied by an induction of apoptotic cell death of the oligodendrocytes (Figure 4C). Expression of CD137L on microglia was essential for activation by CD137-Fc protein and its subsequent ability to induce oligodendrocyte apoptosis as primary CD137L^-/-^ microglia had no effect on oligodendrocyte viability (Figure 4C). Induction of oligodendrocyte apoptosis could also be shown for microglia cell lines N9 and BV-2 upon activation by CD137L engagement (not shown) confirming our hypothesis that CD137L-activated microglia cause oligodendrocyte apoptosis.

### CD137L-activated microglia kills oligodendrocytes via ROS

**Figure 5:**
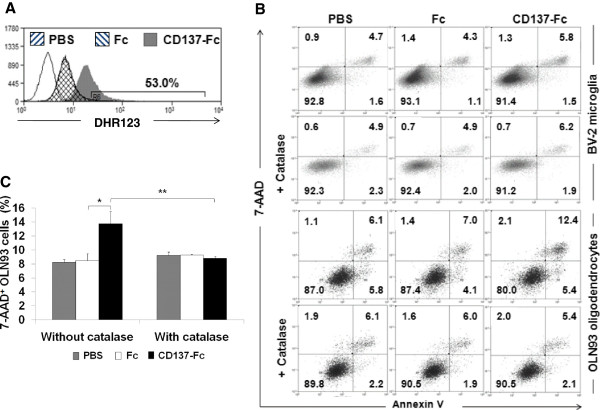
**CD137L-activated microglia induces oligodendrocyte apoptosis via ROS.** (**A**) BV-2 cells were cultured on uncoated plates (PBS) or plates coated with Fc or CD137-Fc protein for 24 h, and were then stimulated with 0.4 μg of PMA for 1 h, stained with DHR123 before production of ROS was quantified by flow cytometry. White histogram: No DHR123. The number in the panel indicates the percentage of positive cells. (**B**) BV-2 cells were co-cultured with OLN93 cells at a 1:1 ratio with or without 10,000 U/mL catalase. The rate of apoptosis of cultures was determined after 24 h by 7-AAD and Annexin V staining. Numbers in quadrants indicate percentages of cells. (**C**) The percentages of 7-AAD^+^ OLN93 cells with and without catalase treatment of B are presented as means ± standard deviations of triplicate measurements. * *P* < 0.05; ** *P* < 0.01.

CD137L-activated monocytes have been shown to induce apoptosis in of T cells when they are in co-culture, and this cell death was mediated by ROS [24]. Therefore, we hypothesized that a similar mechanism may be responsible for the induction of oligodendrocyte death by CD137L-activated microglia. Indeed, CD137L engagement on BV-2 cells induced ROS production (Figure 5A), and when catalase, a hydrogen peroxide scavenger, was added to the co-culture the killing of oligodendrocytes by activated microglia was prevented (Figure 5B). The percentages of late apoptotic and/or dead OLN93 cells for the different conditions are quantitatively depicted in (Figure 5C).

## Discussion

The activating effects of CD137L signals on myeloid cells are well documented [8]. For example, CD137L signaling induces attachment, activation, migration, survival, proliferation, and differentiation in human monocytes [9-13,16]. Since microglia, the main resident myeloid cells in the CNS are similar or identical to tissue macrophages [19,20] it was surmised that the CD137L signal activates microglia, and indeed our results confirm this notion.

More surprising was the fact that the CD137L signal seems to be pivotal for microglia activation since considerably fewer activated microglia were found in the CNS of CD137L^-/-^ mice compared to WT mice during EAE, a neuroinflammatory disease. This result was unexpected since activation of macrophages, and microglia, can be mediated by numerous pathways and is highly redundant.

Microglia has been shown to play a pivotal role in EAE and MS [21,22]. The major pathogenic event in MS is the killing of oligodendrocytes by activated immune cells leading to the loss of axonal myelin sheaths and the subsequent death of the demyelinated neurons. Activated microglia has been demonstrated to induce programmed cell death in oligodendrocytes [23].

The lower microglia activation in the CNS of CD137L^-/-^ mice correlated with a lower number of dying oligodendrocytes during EAE, implying that the microglia caused the oligodendrocyte death. This assumption could indeed be confirmed by demonstrating *in vitro* that CD137L-activated microglia induces oligodendrocyte apoptosis. The pathological relevance of this finding is supported by the fact that both microglia cell lines as well as primary microglia induced the death of oligodendrocytes in response to CD137L signaling. The specificity and essential requirement of CD137L signaling in this process was demonstrated by the inability of CD137L-deficient microglia to induce oligodendrocyte apoptosis.

The induction of oligodendrocyte apoptosis by CD137L-activated microglia occurs via production of ROS. This parallels induction of T cell apoptosis by CD137L-activated monocytes which occurs during the first 24 h of CD137L engagement, and which is thought be a mechanism of infection-induced T cell attrition [24]. Only longer-term CD137L-signaling induces differentiation of monocytes to proinflammatory CD137L-DC [15,16].

In general, the CD137L signal seems to induce a proinflammatory state in myeloid cells as evidenced by proinflammatory cytokine secretion and ROS production in microglia and monocytes. Also, CD137L-DC induce T cells to secrete IFN-γ, IL-13, and IL-17 but to reduce IL-10 [16].

There is a species difference since murine monocytes do not differentiate to DC as do human monocytes in response to CD137L signaling *in vitro*[25]. However, *in vivo* activation of microglia does produce a proinflammatory state. Also, in the murine microglia cell lines BV-2 and N9 a proinflammatory state was induced by CD137L signaling.

CD137 and its ligand have been shown to influence the development of EAE. Agonistic anti-CD137 antibodies administered during the induction phase reduced the incidence and severity of the disease. Potential mechanisms are an increased activation of T cells and a subsequent higher rate of activation induced cell death, as well as a skewing of the T cell response towards regulatory T cells [26,27].

Given these data and our findings that in the absence of CD137L there is less microglia activation and less oligodendrocyte apoptosis one could speculate that EAE induction in CD137L^-/-^ mice may result in a reduced severity. However, the effects of CD137 or CD137L manipulations are very difficult to predict. For example, treatment of tumor-bearing mice with agonistic anti-CD137 mAb enhances the anti-tumor immune responses leading to tumor rejection [28]. Yet treatment with the very same mAb ameliorates collagen-induced arthritis and chronic graft-versus-host disease [29,30]. But it exacerbates acute graft-versus-host disease [31]. The reasons for these unexpected and difficult to reconcile effects are not yet understood. Therefore, we are currently addressing experimentally what influence the absence of CD137L may have on EAE.

Though our data indicate an important contribution of the CD137 receptor/ligand system to neuroinflammatory reactions in the CNS of the mouse it is unknown whether the CD137 receptor/ligand system plays a similar role in the human brain However, we have shown previously that CD137 and CD137L are expressed in the human CNS, and that the expression increases during inflammation caused by mycobacterial infection [32].

This study extends previous work on CD137L signals activating myeloid cells by showing for the first time that CD137L signaling also activates microglia. Further, this study demonstrates an involvement of the CD137 receptor/ligand system in neuroinflammatory conditions suggesting it may also contribute to neurodegenerative diseases.

## Abbreviations

APC, Antigen presenting cells; DC, Dendritic cells; EAE, Experimental autoimmune encephalomyelitis; CD137L, CD137 ligand; MS, Multiple sclerosis; ROS, Reactive oxygen species.

## Authors’ contributions

YAY planned and performed the experiments, analyzed the data, and contributed to writing the manuscript. JMMG and LC performed the EAE induction. SG is the supervisor of LC and participated in the design and discussion of the project. EAL helped in the design of the experiments and participated actively in discussion of the project and editorial work of the manuscript. HS was instrumental to the planning and execution of the project. He wrote the manuscript and is the Principal Investigator. All of the authors have read, contributed to, and approved the final version of the manuscript.

## Competing interests

The authors declare that they have no competing interests.
